# The use of statins was associated with reduced COVID-19 mortality: a systematic review and meta-analysis

**DOI:** 10.1080/07853890.2021.1933165

**Published:** 2021-06-07

**Authors:** Kuan-Sheng Wu, Pei-Chin Lin, Yao-Shen Chen, Tzu-Cheng Pan, Pei-Ling Tang

**Affiliations:** a Department of Internal Medicine, Division of Infectious Diseases, Kaohsiung Veterans General Hospital, Kaohsiung City, ROC; b Faculty of Medicine, School of Medicine, National Yang Ming Chiao Tung University, Taipei, ROC; c Department of Medical Education and Research, Kaohsiung Veterans General Hospital, Kaohsiung City, ROC; d Department of Pharmacy, School of Pharmacy, Kaohsiung Medical University, Kaohsiung City, ROC; e Department of Administration, Kaohsiung Veterans General Hospital, Kaohsiung City, ROC; f Research Center of Medical Informatics, Kaohsiung Veterans General Hospital, Kaohsiung City, ROC; g Department of Health-Business Administration, Fooyin University, Kaohsiung City, ROC; h College of Nursing, Kaohsiung Medical University, Kaohsiung City, ROC

**Keywords:** COVID-19, meta-analysis, mechanical ventilator, mortality, statin, SARS-CoV-2

## Abstract

**Background:**

Statins are widely used to treat people with metabolic and cardiovascular disorders. The effect of statins on coronavirus disease 2019 (COVID-19) is unclear. To investigate the association between statins and COVID-19 outcomes and, if possible, identify the subgroup population that benefits most from statin use.

**Materials and methods:**

A systematic review and meta-analysis of published studies that included statin users and described COVID-19 outcomes through 10 November 2020. This study used the generic inverse variance method to perform meta-analyses with random-effects modelling. The main outcomes were evaluation of the need for invasive mechanical ventilator (IMV) support, the need for intensive care unit (ICU) care and death. All outcomes were measured as dichotomous variables.

**Results:**

A total of 28 observational studies, covering data from 63,537 individuals with COVID-19, were included. The use of statins was significantly associated with decreased mortality (odds ratio [OR] = 0.71, 95% confidence interval [CI]: 0.55–0.92, *I*^2^=72%) and the need for IMV (OR = 0.81, 95% CI: 0.69–0.95, *I*^2^=0%) but was not linked to the need for ICU care (OR = 0.91, 95% CI: 0.55–1.51, *I*^2^=66%). Subgroup analysis further identified five types of studies in which statin users had even lower odds of death.

**Conclusions:**

The use of statins was significantly associated with a reduced need for IMV and decreased mortality among individuals with COVID-19. Statins may not need to be discontinued because of concern for COVID-19 on admission. Further randomized controlled trial (RCTs) are needed to clarify the causal effect between statin use and severe COVID-19 outcomes.

**Key messages**
- Participants in five types of studies were shown to have even lower odds of death when taking statins.
- The use of statins was significantly associated with a reduced need for invasive mechanical ventilation and decreased all-cause mortality among individuals with COVID-19. However, statin use did not prevent participants from needing care in the intensive care unit.
- The results justify performing randomized controlled trials (RCTs) to validate the benefits of statins on COVID-19 outcomes.

## Introduction

Coronavirus disease 2019 (COVID-19) has been a tremendous threat to human life since the pandemic began. People at high risk of unfavourable outcomes caused by COVID-19 include elderly persons and individuals who have chronic metabolic and cardiovascular comorbidities [1,2]. Statins are a class of medications that are widely used to manage metabolic syndrome and cardiovascular diseases. A nationwide report showed that up to 30% of the population aged ≥45 years took statins [3]. Whether the use of statins will affect the clinical course and prognosis of COVID-19 is gaining increasing attention [4,5].

Statins are primarily used to lower serum lipids by inhibiting HMG-CoA reductase. In addition, statins are also known to have pleiotropic effects, including (1) anti-inflammation, (2) immunomodulation, (3) upregulation of angiotensin-converting enzyme 2 (ACE2) receptor expression, (4) antithrombosis and (5) antioxidation [4,6–8]. Some of the effects may have complicated interactions with COVID-19. For example, ACE2 is the main entry receptor of severe acute respiratory syndrome coronavirus 2 (SARS-CoV-2). Chronic use of statins may theoretically increase susceptibility to COVID-19. On the other hand, ACE2 mediated conversion of angiotensin II to angiotensin (1–7) peptide, which can protect the lung from acute injury [6,9,10]. The overall influence of statin use on COVID-19 remains unclear.

An increasing number of researchers are focussing on the relationship between statins and COVID-19 [11–14]. Currently, these studies are all observational, and there are no published randomized controlled trials (RCTs). To date, most studies agree that statins do not have to be discontinued after the onset of COVID-19 [11,13,15]. However, there is still no concrete conclusion about the impact of statins on the prognosis of COVID-19.

This study aims to explore the relationship between statins and COVID-19. Through a comprehensive systematic review and meta-analysis, we expect to answer two clinical questions: (1) will the use of statins lead to a favourable/poor outcome of COVID-19, and (2) can statins decrease the mortality of COVID-19 in a subgroup of statin users? The study results will provide valuable information to health care workers and guide the choice of potential participants in future RCTs.

## Materials and methods

### Search strategy

This study was a systematic review and meta-analysis conducted following the Preferred Reporting Items for Systematic Reviews and Meta-Analyses (PRISMA) recommendations [16] (Supplementary Table S3). We searched the PubMed, Cochrane Library, Embase and MEDLINE Online databases for relevant articles published through 10 November 2020, using the keywords and related MeSH terms as follows: “COVID-19”, “statin’ and “HMG-CoA reductase”. There was no restriction on language or publication status. The reference lists of eligible studies were also manually searched to identify any additional relevant studies. Detailed search strategies are described in Supplementary Table S1.

### Selection criteria

The inclusion criteria for this study are described as follows in the order of population, intervention, comparator, outcome, study design (PICOS) formulation. The population were individuals with positive/confirmed COVID-19; the intervention was the use of statin before or during the hospital course of COVID-19; the comparators were those who did not take statin before or during COVID-19. This study set up three outcomes to evaluate the relationship between statin use and COVID-19, including intensive care unit (ICU) care, use of an invasive mechanical ventilator (IMV) and death. Any study that described at least one of the outcomes was included for assessment and analysis. Eligible study designs included RCT, cohort, clinical trial, case-cohort and cross-over design. The following types of articles were excluded, including review articles, hypothesis, case reports, articles in a non-English language, articles focussing on paediatric populations (17 years of age or younger), articles with a Newcastle–Ottawa scale (NOS) score of 6 or below, articles providing inadequate information and articles not relevant to the study goal. Two investigators (TCP and PLT) independently performed a systematic review using the same criteria and included studies on the basis of agreement. Upon disagreement, a third investigator (PCL) joined and helped make the final decision.

### Data extraction

Eligible studies were first deduplicated by EndNote X9. Then, two investigators (KSW and PLT) independently extracted data from the included studies using an established data collection form. Collected variables included the first author’s name, publication year, study country, study design, sample size, study period, demographics of participants, follow-up duration, diagnostic method of COVID-19, study quality and outcomes. We also contacted corresponding authors to gather missing data when needed. Both raw data and results presented as relative risk/odds ratio (OR)/hazard ratio were included. If a study provided unadjusted and adjusted results, we extracted the adjusted results. The case fatality rate (CFR) of each study was calculated using the number of deceased individuals divided by the number of all individuals [17]. Any discrepancies in data extraction or quality assessment were discussed with a third investigator (TCP) to reach a final agreement.

### Quality and risk of bias assessments

This study used the Cochrane risk of bias tool [18] and NoS [19] to appraise the quality of RCTs and observational studies. The NoS consists of eight items covering three broad perspectives: The selection of the study groups, the comparability of the groups and the ascertainment of either the exposure or outcome of interest. The total maximum score of these three perspectives is nine. A study that scores equal to or higher than seven is considered high-quality research. Besides, a funnel plot was used to evaluate the existence of publication bias.

### Data synthesis and statistical analysis

This study used the generic inverse variance method to perform meta-analysis since some of the enrolled studies reported results with an inverse design. We conservatively chose random effect modelling for analysis since differences in the study population and study design between studies were expected. If an article presented both unadjusted and adjusted results, we retrieved the adjusted results for meta-analysis. This study used the odds ratio to present the overall effect estimates in forest plots and subgroup analysis. The results of the meta-analysis are shown as forest plots with heterogeneity tested by *I*^2^ analysis. An *I*^2^> 50% indicated the existence of substantial heterogeneity, and subgroup analysis was further performed. All meta-analyses were conducted by Review Manager version 5.3 software (Cochrane Collaboration, Oxford, UK) provided by Cochrane. In addition, a funnel plot was generated with Stata Statistical Software version 15.0 (StataCorp LLC, College Station, TX) to evaluate the possibility of publication bias.

## Results

### Selection of study

A total of 502 articles were identified by searching the database and related websites. After deduplication, record screening and eligibility assessment, 28 studies remained for qualitative synthesis, as shown in Table 1 [11,12,14,20–44]. Overall, these 28 selected articles evaluated 86,835 individuals with COVID-19 infection (Supplementary Figure S1). Out of the 28 studies, six studies reported irrelevant outcomes to our study aims [24,26,28,30,31,35]. The last column of Table 1 detailed the outcomes of each study. Finally, 22 studies were eligible for quantitative synthesis [11,12, 14,20–23,25,27,29,32–34,36–44].

**Table 1.** Basic information of the included studies.

Author, year	Study country	Study sample	Enrolled period	Population	Age	Sex (%, male)	Statin regimen	Statin distribution (%, yes)	Comorbidity	Outcomes
Alamdari et al. (2020) [20]	Iran/Tehran	459	30 January–5 April	Hospitalized patients with COVID-19	Mean ± SD: 6.17 ± 11.89	69.7	Drug history	25.5	BMI >35 kg/m^2^, DM, hypertensive disorders, coronary heart disease, CKD, CLD, COPD, malignancy, immunocompromized	Mortality
Argenziano et al. (2020) [21]	USA/New York	1000	1 March–5 April	All patients with COVID-19 who received emergency department or inpatient care	Median (IQR): 63.0 (50.0–75.0)	59.6	Drug history, Inpatient drug treatment	36.1	Hypertension, DM, CAD, CHF, pulmonary disease, asthma, COPD, OSA, interstitial lung disease, renal disease, history of stroke, active cancer, transplant history, rheumatological disease, HIV, viral hepatitis, cirrhosis, obesity (BMI >30 kg/m^2^)	ICU admission
Cariou Bertrand et al. (2020) [22]	French	1317	10 March–10 April	Hospitalized patients with COVID-19	Mean ± SD: 69.8 ± 13.0	64.9	Drug history	47.6	HF, NAFLD or liver cirrhosis, active cancer, COPD, Treated OSA, organ graft, end-stage renal failure	Mortality, primary outcome
Cariou et al. (2020) [14]	French	2449	10 March–10 April	Hospitalized patients with COVID-19	Mean ± SD: 70.9 ± 12.5	64.03	Drug history	48.67	HF, NAFLD, liver cirrhosis, active cancer, COPD, treated OSA	IMV, mortality
Daniels et al. (2020) [23]	USA/California	170	10 February–17 June	Hospitalized patients with COVID-19	Mean ± SD: 59 ± 19	58	Drug history	27.1	Obesity, DM, hypertension, CVD, HF, stroke, CKD, asthma, COPD, cancer, HIV	Mortality, severity, ICU admission
Davoudi-Monfared et al. (2020) [24]	Iran/Tehran	81	29 February–3 April	Patients with severe COVID-19	Mean ± SD IFN groups: 56.0 ± 16 control group: 59.5 ± 14	54.3	Inpatient drug treatment	18.52	Any comorbidity, hypertension, DM, ischaemic heart disease, endocrine disorder, malignancy, neuropsychiatric disorders, haematologic disorder, rheumatoid disorder, renal disease, liver disease, rheumatoid arthritis, asthma, transplantation, COPD	IFN
De Spiegeleer et al. (2020) [25]	Belgium	154	1 March–16 April	All (anonymized) residents at 2 care homes with COVID-19	Mean ± SD: 85.9 ± 7.2	33.1	Drug history	21	Hypertension, cardiovascular disease, COPD, DM, renal failure, liver disease, obesity	Severity
Dreher et al. (2020) [26]	Germany/Aachen	50	February–March	Hospitalized patients with COVID-19	Median (IQR): 65 (58–76)	66	Drug history	36	Arterial hypertension, obesity, overweight, DM, prediabetes, COPD, OSA, bronchial asthma, other pulmonary diseases, CKD, nicotine abuse, cerebral arterial occlusive disease, cancer, chronic hepatitis, chronic liver failure, PAD	ARDS
Grasselli et al. (2020) [27]	Italy	3988	20 February–22 April	Patients with confirmed COVID-19 infection admitted to one of the network ICUs	Median (IQR): 63 (56–69)	79.9	Drug history	29.79	Hypertension, hypercholesterolaemia, heart disease, DM, malignant neoplasm, COPD, CKD, liver disease, other disease	Mortality
Gupta et al. (2020) [12]	USA/New York	1296^a^	1 February–12 May	COVID-19 positive patients	Median (IQR): 69 (61 − 77)	56.48	Drug history	50	Hypertension, DM, CAD, HF, chronic lung disease, CKD, stroke/TIA, atrial arrhythmias, liver disease	IMV, Mortality
Higuchi et al. (2021) [28]	Japan	57	20 February–10 June	Consecutive hospital- admitted patients with COVID-19	Median (IQR): 52 (35–69.5)	56.1	Drug history	21.1	Hypertension, cardiovascular diseases, COPD, asthma, DM, hyperlipidaemia, CKD, haemodialysis, solid tumour	Disease status
Israel et al. (2020) [29]	Israel	37,212^a^	25 September–10 October	Hospital admission and confirmation cases	Mean ± SD hospitalized: 56.8 ± 18.9 not hospitalized: 57.2 ± 18.7	49.11	Drug history	Cohort 1: 4.81	Arrhythmia, asthma, CHF, COPD, DM, hypertension, ischaemic heart disease, malignancy, CKD, obesity	Mortality
Jakob et al. (2021) [30]	Germany	2155	16 March–14 May	COVID-19 positive patients	NA	59.7	Drug history	23.2	Cardiovascular disease, DM, pulmonary disease, haematological and/or oncological disease, neurological disease, kidney disease, connective tissue disease, peptic ulcer disease, CLD, liver cirrhosis, organ transplantation, rheumatic disease, HIV/AIDS	Definition complicated clinical staging
Mallow et al. (2020) [31]	USA	21,676	15 March–30 April	COVID-19 positive patients	Mean ± SD: 64.9 ± 17.2	52.8	Inpatient drug treatment	24.51	Chronic lung disease, moderate-to-severe asthma, severe heart disease, immunocompromized, obesity, diabetes, CKD with dialysis, liver disease, hypertension, CKD (any stage), haemoptysis, hypothyroidism, DNR status, MI, CHF, cerebrovascular disease, dementia, chronic pulmonary disease, connective tissue disease, peptic ulcer disease, mild liver disease, DM without end-organ damage, DM with end-organ damage, hemiplegia, moderate or severe renal disease tumour without metastases, moderate or severe liver disease, metastatic solid tumour, AIDS	CDC risk factors
McCarthy et al. (2020) [32]	USA/Boston	247	7 March–30 March	Hospitalized with confirmed SARS-CoV-2	Median (IQR): 61 (50–76)	57.6	Drug history	75.7	Overweight, obesity, overweight or obesity, asthma, COPD, interstitial lung disease, OSA/OHS, hypertension, hyperlipidaemia, DM, known CAD, prior MI, prior revascularization, HF, PAD, prior stroke/TIA, AF, liver cirrhosis, CKD, CKD on dialysis, history of malignancy, active malignancy, prior organ transplant, rheumatologic or inflammatory disorder requiring immunosuppression	ICU admission
Nguyen et al. (2020) [33]	USA	689	16 March–16 April	COVID-19 positive patients	Median (IQR): 55 (40–68)	43	Drug history	16.7	Obesity, DM, hypertension, stroke/cerebrovascular	Mortality
Nicholson et al. (2021) [34]	USA	1042	19 May– 20 July	Patients with laboratory-confirmed COVID-19 infection	Median (IQR): 64 (53–75)	56.81	Drug history	49.04	DM, CKD (stages III–VI), COPD, asthma, cancer, CAD, HF, AF	IMV, mortality
Pitscheider et al. (2020) [35]	Austria	609	N/A	Hospitalized patients with COVID-19	Median (IQR) SARS-CoV-2 cohort: 68 (54–79)	56.65	Drug history	11.99	CAD, arterial hypertension, DM, COPD	Comparison of COVID-19 patients and influenza patients
Rodriguez-Nava et al. (2020) [36]	USA/Evanston	87	March–May	Laboratory-confirmed COVID-19 admitted to the community hospital ICU	Median (IQR): 68.0 (58.0–75.0)	64.4	Inpatient drug treatment	54	Hypertension, cardiovascular disease	Mortality
Rossi et al. (2020) [37]	Italy	71	29 February–20 May	COVID-19 positive patients	Median (IQR): 71 (64–92)	56.33	Drug history	59.1	DM, CAD, hypertension, hyperlipidaemia, CKD, COPD, cancer	Mortality
Saeed et al. (2020) [38]	USA/New York	4252	1 March–2 May	Hospital admission and confirmation cases	Mean ± SD: 65 ± 16	53.50	Inpatient drug treatment	31.87	DM, hypertension, ASHD, lung disease	Mortality
Song et al. (2020) [39]	USA/Rhode Island	249	17 March–10 April	Patients with laboratory confirmed COVID-19	Median (IQR): 62.0 (51.0–75.0)	57	Drug history	49.4	Hypertension, DM, hypercholesterolaemia, obesity (BMI > 30 kg/m^2^), CAD, cerebrovascular disease, aortic or mitral valvulopathy, CHF, History of pulmonary embolism, COPD, chronic renal failure	IMV, ICU admission, mortality
Tan et al. (2020) [40]	Singapore	717	22 January–15 April	Hospitalized patients with COVID-19	Median (IQR): 46 (19–57)	57.18	Drug history	21.06	High cardiovascular risk profile, hypertension, hyperlipidaemia, DM, previous atherosclerotic complications, CHF, lung disease, CKD, history of other malignancy	IMV, ICU admission, mortality
Wang et al. (2020) [41]	USA/New York	58	1 March–30 April	COVID-19 positive patients	Median (IQR): 67 (12.5)	52	Drug history	47	Low function, DM, hypertension	Mortality
Yan et al. (2020) [42]	China/Zhejiang	578	10 January–28 February	COVID-19 positive patients	Mean ± SD: 49.18 ± 14.16	50.7	Drug history	2.6	DM, hypertension, cardiovascular diseases, renal Failure	Severity
Yang et al. (2020) [43]	China/Wuhan	836	1 January–23 March	COVID-19 positive patients	Median (IQR): 73.5 (64.5–86.6)	48.44	Inpatient drug treatment	25.64	HBV, DM, hypertension, cardio- or cerebrovascular, tumour	Mortality
Zenga et al. (2020) [44]	China/Wuhan	1031	27 January–8 March	Hospitalized with COVID-19	Mean ± SD: 60.3 ± 14.3	52.2	Drug history	3.6	Hypertension, DM, cardiovascular disease, nervous system disease, chronic lung disease, tumour	Mortality
Zhang et al. (2020) [11]	China/Hubei	4305^a^	30 December 2019–17 April 2020	Hospitalized patients with COVID-19	Median (IQR): 66.0 (59.0–72.0)	48.85	Inpatient drug treatment	8.72	COPD, hHypertension, DM, CHD, stroke, cancer	IMV, ICU admission, mortality

AF: atrial fibrillation; ARDS: acute respiratory distress syndrome; ASHD: arteriosclerotic heart disease; BMI: body mass index; CT: computed tomography; CLD: chronic liver disease; CHD: congenital heart defect; CAD: coronary artery disease; CKD: chronic kidney disease; CHF: congestive heart failure; COPD: chronic obstructive pulmonary disease; DM: diabetes mellitus; HIV: human immunodeficiency virus; HBV: hepatitis B virus; HF: heart failure; IMV: invasive mechanical ventilator; ICU: intensive care unit; IFN: interferon; MI: myocardial infarction; OSA: obstructive sleep apnoea; OHS: obesity hypoventilation syndrome; PAD: peripheral artery disease; RT-PCR: real-time reverse-transcriptase polymerase chain reaction; SD: standard deviation; TIA: transient ischaemic attack; USA: United States of America. ^a^Number of participants after applying propensity score-matching model to minimize differences in baseline characteristics between statin users *versus* non-statin users.

### Characteristics of selected studies

All 28 articles included for qualitative synthesis were observational studies. The participants were individuals who were confirmed to have contracted COVID-19, were hospitalized for management and underwent a follow-up period of at least 28 d after study entry. Among these selected studies, 11 were conducted in the United States (USA) [12,21,23,31–34,36,38,39,41], 9 in Asia [11,20,24,28,29,40,42–44] and 8 in Europe [14,22,25–27,30,35,37]. The studies’ sample sizes varied from 50 to 37,212. The proportion of statin uses ranged from 2% to 75%. Detailed characteristics of the included studies are presented in Table 1. All the studies were of high (*n* = 26) [11,12,14,20,21,23–36,38–44] or moderate quality (*n*= 2) [22,37] by the NoS assessment. The most biased items were the comparability of groups and ascertainment of nonresponse rate (Supplementary Table S2). The funnel plot showed an asymmetrical distribution of the points, suggesting a lack of publication bias (*p* value for Egger’s test = 0.9933; *p* value for Begg’s test = 0.4767) (Supplementary Figure S2).

### Statins and severe outcomes of COVID-19

**Figure 1.**
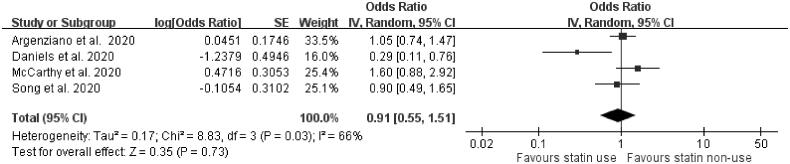
Forest plot showing the association between statin use and the need for intensive care unit care. The squares and bars represent the mean values and 95% CIs of the effect sizes, the area of the squares reflects the weight of the studies, and diamonds represent the combined effects.

**Figure 2.**
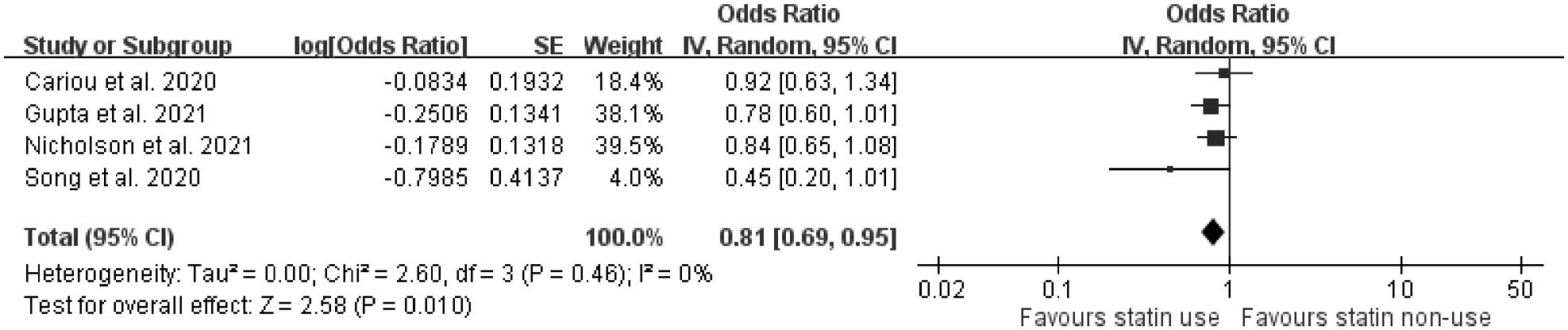
Forest plot showing the association between statins and the need for invasive mechanical ventilator support. The squares and bars represent the mean values and 95% CIs of the effect sizes, the area of the squares reflects the weight of the studies, and diamonds represent the combined effects.

Four articles, two presenting unadjusted outcomes [21,32] and another two presenting adjusted outcomes [23,39], reported the association between statins and ICU care. The meta-analysis showed that the use of statins was not significantly associated with the need for ICU care (OR = 0.91, 95% confidence interval [CI]: 0.55–1.51, *p*=.73, *I*^2^= 66%) (Figure 1). Four studies were included to evaluate how statin use affects the need for IMV in COVID-19-confirmed individuals. Three of these four studies presented adjusted outcomes regarding IMV use [12,14,39]. The use of statins was significantly associated with a decreased risk of the need for IMV support (OR = 0.81, 95% CI: 0.69–0.95, *p*=.010, *I*^2^=0%) (Figure 2).

### Statins and COVID-19 mortality

**Figure 3.**
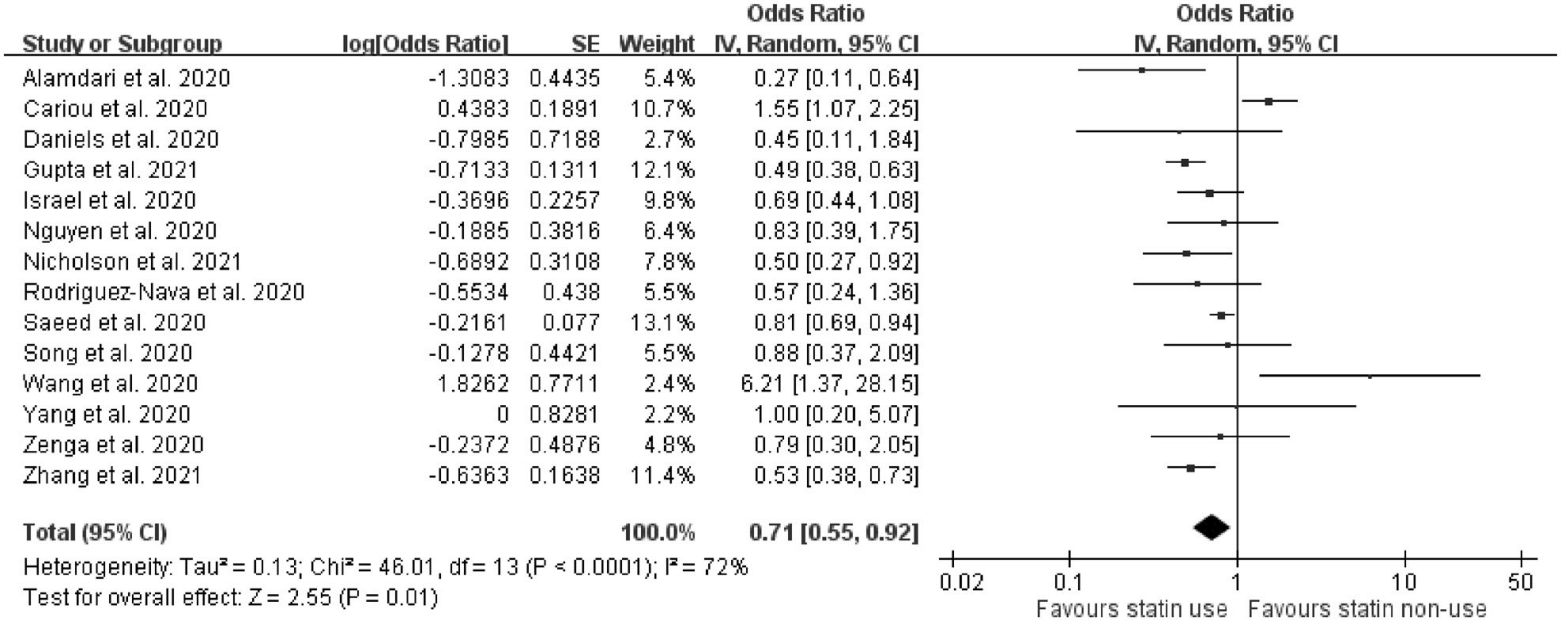
Forest plot showing the association between statins and mortality. The squares and bars represent the mean values and 95% CIs of the effect sizes, the area of the squares reflects the weight of the studies and diamonds represent the combined effects.

Eighteen studies described the mortality outcomes. Among the 18 studies, four were excluded due to incomplete data reporting [22,27,37,40], low quality [22,37] and a focus on short-term mortality (<7 d) only [22,27,40]. A total of 14 articles were included in the final meta-analysis. Seven of these 14 articles used either propensity-matched scores or logistic regression methods to present adjusted outcomes [11,12,14,23,29,34,39], and another seven studies provided unadjusted data [20,33,36,38,41,43,44]. The pooled analysis results revealed that the use of statins was linked to a decrease in the mortality rate of COVID-19 (OR = 0.71, 95% CI: 0.55–0.92, *p*= .01, *I*^2^=72%) (Figure 3).

### Subgroup analysis of statins and COVID-19 mortality

Since substantial heterogeneity existed in the meta-analysis of statins and COVID-19 mortality (*I*^2^=72%), we performed subgroup analysis to explore the source of heterogeneity. We identified five subgroups of studies in which statin users had even lower odds of death, including studies that (1) enrolled participants not limited to those diabetes mellitus (DM) only (OR = 0.64, 95% CI: 0.51–0.81, *p*<.0002, *I*^2^= 58%), (2) had a CFR of lower than 20% (OR = 0.58, 95% CI: 0.41–0.83, *p*=.003, *I*^2^= 26%), (3) presented adjusted outcomes (OR = 0.58, 95% CI: 0.37–0.94, *p*=.03, *I*^2^=0%), (4) were conducted in Asia (OR = 0.57, 95% CI: 0.43–0.75, *p* <.0001, *I*^2^=14%) and (5) emphasized that the timing of statin use was during hospitalization (OR = 0.55, 95% CI: 0.41–0.73, *p*<.0001, *I*^2^=0%). The heterogeneity in these five subgroups of studies was lower than that before the subgroup analyses. On the other hand, one single study that included only individuals with DM revealed that statin use was associated with increased COVID-19 mortality (OR = 1.55, 95% CI: 1.07–2.25, *p*=.02). Table 2 describes the detailed results of the subgroup analyses.

**Table 2.** Subgroup analysis for COVID-19 mortality.

Subgroup	Statin (n)	Paper (n)	Relative risk (95% CI)	*I* ^2^	*p*	*p* (interaction between groups)	*I*^2^ (heterogeneity between groups)
DM						<.0001	93.6
Enrolled only individuals with DM [14]	1192	1	1.55 (1.07–2.25)	N/A	.02		
Enrolled individuals with and without DM [11,12,20,23,29,33,34,36,38,39,41,43,44]	5663	13	0.64 (0.51–0.81)	58	.0002		
CFR						.19	40.7
≤20 [11,20,33,39,44]	1229	5	0.58 (0.41–0.83)	26	.003		
>20 [12,14,34,36,41,43]	3789	7	0.84 (0.55–1.26)	83	.39		
Different statistical methods						.70	0
Crude odds ratio [20,33,36,38,41,43,44]	1684	7	0.77 (0.49–1.20)	56	.24		
Adjusted odds ratio [23,34,39]	679	3	0.58 (0.37–0.94)	0	.03		
Match [11,12,14,29]	4492	4	0.72 (0.43–1.20)	89	.21		
Geographic location of studies						.13	57.3
Asia [11,20,29,43,44]	2817	5	0.57 (0.43–0.75)	14	<.0001		
` Non-Asian [12,14,23,33,34,36,38,39,41]	4038	9	0.80 (0.57–1.14)	78	.22		
Drug treatment timing						.15	51.9
History of drug treatment [12,14,20,23,29,33,34,38,39,41,44]	5937	11	0.75 (0.55–1.02)	76	.06		
Inpatient drug treatment [11,36,43]	918	3	0.55 (0.41–0.73)	0	<.0001		

CFR: case fatality rate; DM: diabetes mellitus

The measure of mortality outcome is provided for each subgroup, and the interaction *p* value and *I*^2^ are provided for subgroup differences. All analyses were conducted using inverse variance and a random-effects model.

## Discussion

Our study demonstrated that the use of statins was associated with a reduced mortality risk of COVID-19. When excluding one study enrolling only individuals with DM [14], the association between statin use and COVID-19 mortality was significant. In addition, the subgroup analysis showed that statin use prevented more deaths in studies with a CFR below 20% than in those with a CFR above 20%. This result implies that the benefit of statins seems to be greater when the COVID-19 mortality rate in the community is reduced. Our meta-analyses also revealed that statin use was linked to a reduced need for IMV but could not prevent individuals from needing ICU admission.

Our finding that statins were associated with reduced COVID-19 mortality confirmed the results from previous reports [15,45,46]. Compared with non-statin users, statin users are usually older and have more comorbidities, which both lead to an increased risk of COVID-19 mortality [11,12]. Thus, when the meta-analysis pooled the adjusted and unadjusted results, the association between statin use and mortality might not be obvious because the protective effect of statins might be countered by increased risks from host factors. In our study, statins showed benefits even when the meta-analysis pooled both the adjusted and unadjusted results, making the results more robust and convincing.

One large meta-analysis performed a comprehensive systematic review and concluded statins were not associated with reduced mortality of COVID-19. However, the *I*^2^ of the forest plot demonstrating the association of statin with mortality was as high as 90%, indicating the substantial heterogeneity across the included articles [47]. Therefore, one essential goal of our study was to find out the subgroups that had lower heterogeneity. After the subgroup analysis, this study identified five subgroups in which statins were associated with an even lower COVID-19 mortality. The five subgroups also had a lower heterogeneity than overall participants. Our results may be helpful for participant selection in future RCTs.

One of our subgroup analyses showed that statins had survival benefits in studies with an overall CFR of less than 20% but not in those with a CFR above 20%. There are three possible explanations. First, the beneficial effect of statins was mild. When an individual bears a high risk of COVID-19 mortality, the statins’ impact might be overwhelmed by the host factor and turn out to be statistically nonsignificant. Second, the effect of statins was influenced by competing medical issues. A high overall mortality rate often reflects a more severe epidemic in the community because only individuals with serious conditions can be admitted for treatment. Under such circumstances, medical resources may be insufficient. The health care system may sort medical help to those who had a better prognosis than to those who had the most severe illness. Statin users, often with more comorbidities, may have poorer outcomes than those with sufficient medical resources. Third, all confirmed COVID-19 individuals’ average mortality rate in the USA was 1.82% by 6 March 2021 [48]. The higher the overall mortality rate is in a study, the lower the generalizability of the result is. As more people acquire immunity against SARS-CoV-2, either by vaccination or infection, and the health care system gains more experience in treating COVID-19, we can expect a lower overall mortality rate in the future. We believe that the analysis results focussing on low CFR studies may better apply to future conditions.

According to the study results, statins were associated with a reduced need for mechanical ventilators but did not influence the possibility of ICU admission. Both of the above results had low heterogeneity. Two possible reasons may explain that. First, the criteria for the use of mechanical ventilators or ICU care might vary among different areas, hospitals and even physicians. Insufficient medical resources could also play a role in some cohorts. Second, statins provide lung protection and thus decrease the need for IMV. However, statins could not reduce other nonpulmonary events, such as cardiovascular complications or sepsis. The mechanism of lung protection is most likely *via* upregulation of ACE2 expression, leading to increased angiotensin (1–7) production.

Although one of our subgroup analyses showed that in-hospital use of statins had a greater benefit, we cannot interpret that to mean that starting statins on admission to treat COVID-19 was helpful. According to clinical experiences, most in-hospital statin users were already taking statins before contracting COVID-19. Besides, the majority of articles included in our study emphasized the use of statin was prior to COVID-19 infection, and the results showed statin had a trend of protective effect from getting COVID-19. Retrospective observational studies had an intrinsic limitation in recording clear-cut timing of statin use among a large group of participants. However, our results at least confirmed that statin users can continue taking the medication during COVID-19 infection.

There are several limitations to the study. First, all articles included in our meta-analyses were observational study designs. The baseline demographics of statin users *vs.* nonusers exhibited substantial differences. Furthermore, some studies reported only crude odds ratios, which might lead to an even more significant bias. Second, the type, dose and duration of statins were by no means identical across studies. The majority of the studies did not detail the prescriptions of statins. Thus, even though the current evidence showed that statins might be beneficial for patients with COVID-19, it was almost impossible to provide a clear suggestion about the drug name, dose and duration. Third, a substantial portion of participants in this study had comorbidities of diabetes or cardiovascular diseases, and might have taken other concomitant medication, such as metformin, ACE inhibitors and angiotensin II receptor blockers (ARB). Metformin has shown to be associated with reduced COVID-19 mortality [49], and ACEI/ARB also had close relationship with COVID-19 [50]. The influence of interaction between statins and co-medications on COVID-19 remains unclear and might impact the estimate of statins’ effect. Future RCTs are needed to solve the limitations mentioned above.

## Conclusions

Our results demonstrated that the use of statins was associated with a reduced need for IMV and mortality rate among individuals with COVID-19. Subgroup analyses further identified individuals enrolled in studies with a lower mortality rate who benefitted more from statins. This study also confirmed that statins could be safely used during COVID-19 hospitalization.

## Supplementary Material

Supplemental Material

## Data Availability

The data that support the findings of this study are available from the corresponding author, [Pei-Ling Tang], upon reasonable request.
