# Supplementary material for: The use of statins was associated with reduced COVID-19 mortality: a systematic review and meta-analysis
Source: Ann Med. 2021 Jun 7;53(1):874–84. doi: 10.1080/07853890.2021.1933165 (PMC8189130; doi:10.1080/07853890.2021.1933165)
Supplement: Supplemental Material [file IANN_A_1933165_SM2161.docx]

**Supplementary Materials
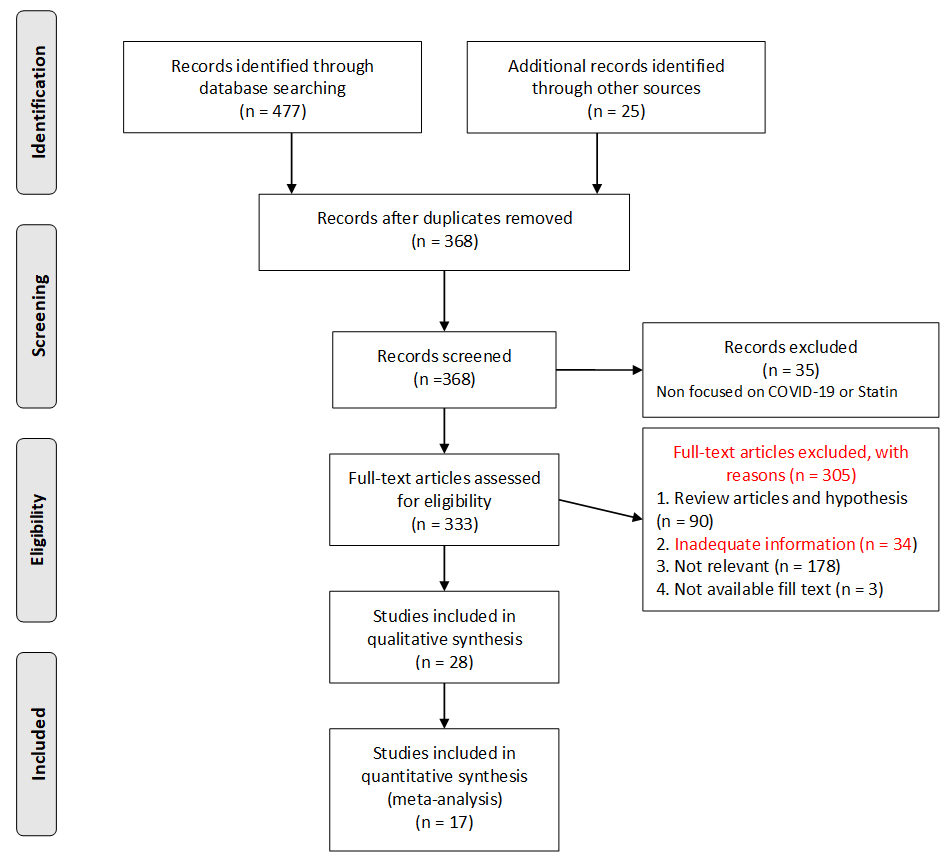
**

**Figure S1.** Flow diagram

The flow diagram demonstrates the study selection process in the systematic review and meta-analysis.


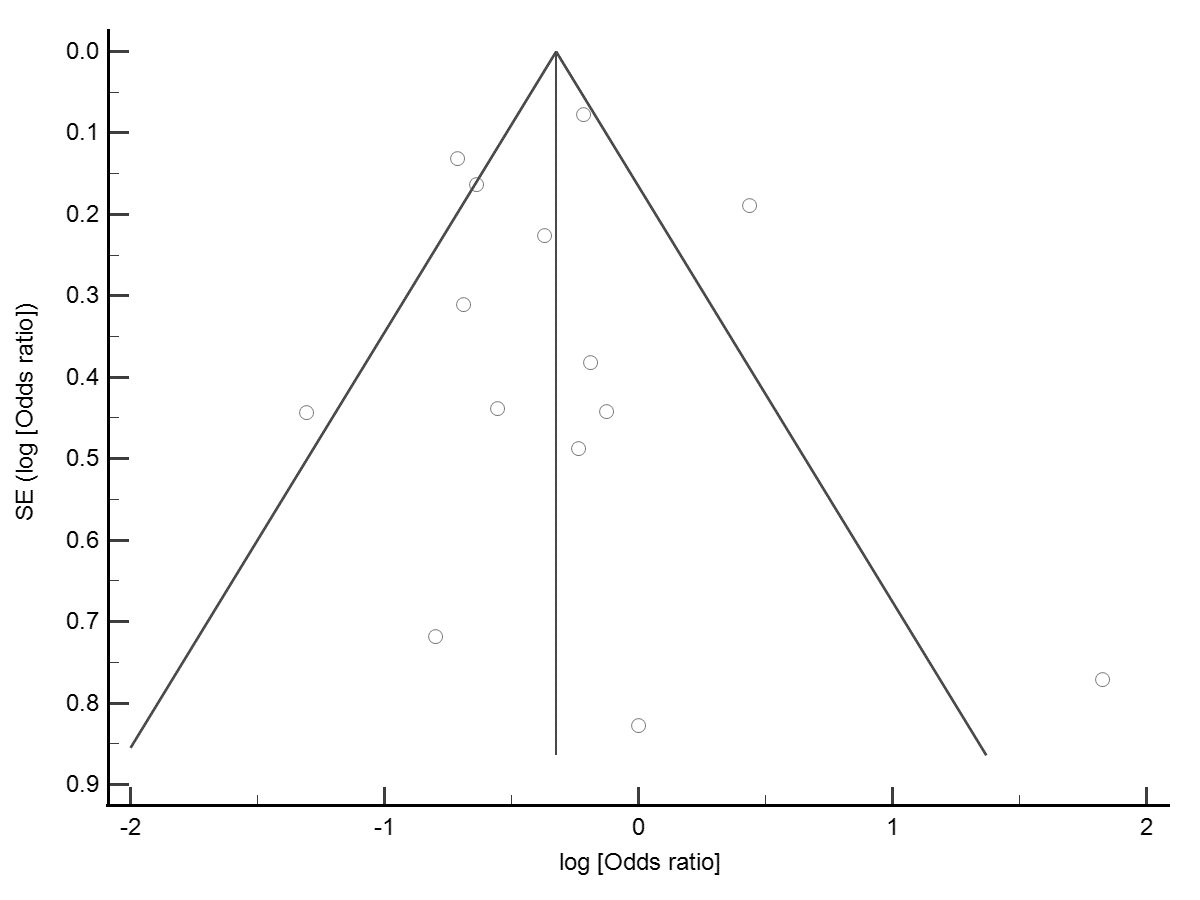


**Figure S2.** Funnel plot for the risk of mortality from observational cohort studies with Statin therapy.

**Publication bias**

| Egger's test | |
| --- | --- |
| Intercept | -0.007225 |
| 95% CI | -1.8366 to 1.8221 |
| Significance level | *p* = .9933 |
| Begg’s test | |
| Kendall's Tau | 0.1429 |
| Significance level | *p* = .4767 |

**Table S1. Search Strategies**

**PubMed**

1. **"2019 novel coronavirus disease"[All Fields] OR "COVID19"[All Fields] OR "COVID-19 pandemic"[All Fields] OR "SARS-CoV-2 infection"[All Fields] OR "COVID-19 virus disease"[All Fields] OR "2019 novel coronavirus infection"[All Fields] OR "2019-nCoV infection"[All Fields] OR "coronavirus disease 2019"[All Fields] OR "coronavirus disease-19"[All Fields] OR "2019-nCoV disease"[All Fields] OR "COVID-19 virus infection"[All Fields] OR "Wuhan seafood market pneumonia virus"[All Fields] OR ("sars cov2"[All Fields] AND "2019"[All Fields]) OR ("covid 19 drug treatment"[Supplementary Concept] OR "covid 19 drug treatment"[All Fields]) OR "Covid-19 treatment"[All Fields] OR "treatment of Covid-19 virus infection"[All Fields] OR "covid 19"[MeSH Terms] OR "sars cov 2"[MeSH Terms] OR "coronavirus infections" [MeSH Terms]**
2. **"statin treatment"[All Fields] OR "coenzyme Q10"[All Fields] OR "statin"[All Fields] OR "HMG‐CoA reductase inhibitors"[All Fields] OR "Simvastatin"[All Fields] OR "Lovastatin"[All Fields] OR "Fluvastatin"[All Fields] OR "Pravastatin"[All Fields] OR "Rosuvastatin"[All Fields] OR "Atorvastatin"[All Fields] OR "statins"[All Fields] OR "lipid-lowering therapy"[All Fields] OR "dyslipidemia therapy"[All Fields] OR "pitavastatin"[All Fields] OR "anticholesteremic agents"[All Fields] OR "cerivastatin"[All Fields] OR "HMG-CoA reductase inhibitor"[All Fields] OR "HMGCoA RI"[All Fields] OR "HMG CoA Statin"[All Fields] OR "HMG CoA Statins"[All Fields] OR "HMG-CoA Statin"[All Fields] OR "HMG-CoA Statins"[All Fields] OR "HMG CoA Reductase Inhibitor"[All Fields] OR "HMG CoA Reductase Inhibitors"[All Fields] OR "HMG-CoA Reductase Inhibitors"[All Fields] OR "Hydroxymethylglutaryl CoA Inhibitor"[All Fields] OR "Hydroxymethylglutaryl CoA Inhibitors"[All Fields] OR "Hydroxymethylglutaryl-CoA Inhibitor"[All Fields] OR "Hydroxymethylglutaryl-CoA Inhibitors"[All Fields] OR "Hydroxymethylglutaryl Coenzyme A Inhibitor"[All Fields] OR "Hydroxymethylglutaryl Coenzyme A Inhibitors"[All Fields] OR "Hydroxymethylglutaryl-Coenzyme A Inhibitor"[All Fields] OR "Hydroxymethylglutaryl-Coenzyme A Inhibitors"[All Fields] OR "hydroxymethylglutarylcoenzyme A reductase inhibitors"[All Fields] OR "Hydroxymethylglutaryl CoA Reductase Inhibitor"[All Fields] OR "Hydroxymethylglutaryl CoA Reductase Inhibitors"[All Fields] OR "Hydroxymethylglutaryl-CoA Reductase Inhibitor"[All Fields] OR "Hydroxymethylglutaryl-CoA Reductase Inhibitors"[All Fields] OR "HMGCR inhibitor"[All Fields] OR "HMGCR inhibitors"[All Fields] OR " lipid-lowering drugs"[All Fields] OR "lipid-lowering agents"[All Fields] OR "hydroxymethylglutaryl coa reductase inhibitors"[MeSH Terms]**
3. **1 AND 2**

**Cochrane Library**

ALL TEXT

1. ((2019 novel coronavirus disease) or (COVID19) or (COVID-19 pandemic) or (SARS-CoV-2 infection) or (COVID-19 virus disease) or (2019 novel coronavirus infection) or (2019 nCoV infection) or (coronavirus disease 2019) or (coronavirus disease-19) or (2019 nCoV disease) or (COVID-19 virus infection) or (Wuhan seafood market pneumonia virus) or (SARS-Cov2 2019) or (coronavirus disease 2019 drug treatment) or (Covid-19 treatment) or (treatment of Covid-19 virus infection)) or (SARS-CoV) OR (SARS Virus) or (severe acute respiratory syndrome) or (SARS) or (SARS Coronavirus) or (SARS-Associated Coronavirus) or (SARS-Related Coronavirus) or (Severe Acute Respiratory Syndrome Virus) or (Severe acute respiratory syndrome-related coronavirus) or (Urbani SARS-Associated Coronavirus) or (Respiratory Syndrome, Acute, Severe) or (Respiratory Syndrome, Severe Acute)) or (Coronavirus Infections) or (MERS) or (Middle East respiratory syndrome) or (MERS-CoV) or (MERS-CoV infections) or (MERS Virus) or (Middle East respiratory syndrome-related coronavirus) or (Middle East Respiratory Syndrome Coronavirus))
2. ((statin treatment) or (coenzyme Q10) or (statin) or (HMG?CoA reductase inhibitors) or (Simvastatin) or (Lovastatin) or (Fluvastatin) or (Pravastatin) or (Rosuvastatin) or (Atorvastatin) or (statins) or (lipid-lowering therapy) or (dyslipidemia therapy) or (pitavastatin) or (anticholesteremic agents) or (cerivastatin) or (HMG-CoA reductase inhibitor) or (HMGCoA RI) or (HMG CoA Statin) or (HMG CoA Statins) or (HMG-CoA Statin) or (HMG-CoA Statins) or (HMG CoA Reductase Inhibitor) or (HMG CoA Reductase Inhibitors) or (HMG-CoA Reductase Inhibitors) or (Hydroxymethylglutaryl CoA Inhibitor) or (Hydroxymethylglutaryl CoA Inhibitors) or (Hydroxymethylglutaryl-CoA Inhibitor) or (Hydroxymethylglutaryl-CoA Inhibitors) or (Hydroxymethylglutaryl Coenzyme A Inhibitor) or (Hydroxymethylglutaryl Coenzyme A Inhibitors) or (Hydroxymethylglutaryl-Coenzyme A Inhibitor) or (Hydroxymethylglutaryl-Coenzyme A Inhibitors) or (hydroxymethylglutarylcoenzyme A reductase inhibitors) or (Hydroxymethylglutaryl CoA Reductase Inhibitor) or (Hydroxymethylglutaryl CoA Reductase Inhibitors) or (Hydroxymethylglutaryl-CoA Reductase Inhibitor) or (Hydroxymethylglutaryl-CoA Reductase Inhibitors) or (HMGCR inhibitor) or (HMGCR inhibitors) or (lipid-lowering drugs) or (lipid-lowering agents))
3. (#1) and (#2)

**MEDLINE Online**

1. (2019 novel coronavirus disease or COVID19 or COVID-19 pandemic or SARS-CoV-2 infection or COVID-19 virus disease or 2019 novel coronavirus infection or 2019-nCoV infection or coronavirus disease 2019 or coronavirus disease-19 or 2019-nCoV disease or COVID-19 virus infection or Wuhan seafood market pneumonia virus or SARS-Cov2 2019 or coronavirus disease 2019 drug treatment or Covid-19 treatment or treatment of Covid-19 virus infection).mp. [mp=title, abstract, original title, name of substance word, subject heading word, floating sub-heading word, keyword heading word, organism supplementary concept word, protocol supplementary concept word, rare disease supplementary concept word, unique identifier, synonyms]
2. (statin treatment or coenzyme Q10 or statin or HMG?CoA reductase inhibitors or Simvastatin or Lovastatin or Fluvastatin or Pravastatin or Rosuvastatin or Atorvastatin or statins or lipid-lowering therapy or dyslipidemia therapy or pitavastatin or anticholesteremic agents or cerivastatin or HMG-CoA reductase inhibitor or HMGCoA RI or HMG CoA Statin or HMG CoA Statins or HMG-CoA Statin or HMG-CoA Statins or HMG CoA Reductase Inhibitor or HMG CoA Reductase Inhibitors or HMG-CoA Reductase Inhibitors or Hydroxymethylglutaryl CoA Inhibitor or Hydroxymethylglutaryl CoA Inhibitors or Hydroxymethylglutaryl-CoA Inhibitor or Hydroxymethylglutaryl-CoA Inhibitors or Hydroxymethylglutaryl Coenzyme A Inhibitor or Hydroxymethylglutaryl Coenzyme A Inhibitors or Hydroxymethylglutaryl-Coenzyme A Inhibitor or Hydroxymethylglutaryl-Coenzyme A Inhibitors or hydroxymethylglutarylcoenzyme A reductase inhibitors or Hydroxymethylglutaryl CoA Reductase Inhibitor or Hydroxymethylglutaryl CoA Reductase Inhibitors or Hydroxymethylglutaryl-CoA Reductase Inhibitor or Hydroxymethylglutaryl-CoA Reductase Inhibitors or HMGCR inhibitor or HMGCR inhibitors or lipid-lowering drugs or lipid-lowering agents).mp. [mp=title, abstract, original title, name of substance word, subject heading word, floating sub-heading word, keyword heading word, organism supplementary concept word, protocol supplementary concept word, rare disease supplementary concept word, unique identifier, synonyms]
3. 1 and 2

**Embase**

1. '2019 novel coronavirus disease' OR 'covid19'/exp OR 'covid19' OR 'covid-19 pandemic' OR 'sars-cov-2 infection' OR 'covid-19 virus disease' OR '2019 novel coronavirus infection' OR '2019-ncov infection'/exp OR '2019-ncov infection' OR 'coronavirus disease 2019'/exp OR 'coronavirus disease 2019' OR 'coronavirus disease-19' OR '2019-ncov disease'/exp OR '2019-ncov disease' OR 'covid-19 virus infection' OR 'wuhan seafood market pneumonia virus'/exp OR 'wuhan seafood market pneumonia virus' OR 'sars-cov2 2019' OR 'coronavirus disease 2019 drug treatment' OR 'covid-19 treatment' OR 'treatment of covid-19 virus infection'
2. 'statin treatment' OR 'coenzyme q10' OR 'statin' OR 'hmg?coa reductase inhibitors' OR 'simvastatin' OR 'lovastatin' OR 'fluvastatin' OR 'pravastatin' OR 'rosuvastatin' OR 'atorvastatin' OR 'statins' OR 'lipid-lowering therapy' OR 'dyslipidemia therapy' OR 'pitavastatin' OR 'anticholesteremic agents' OR 'cerivastatin' OR 'hmg-coa reductase inhibitor' OR 'hmgcoa ri' OR 'hmg coa statin' OR 'hmg coa statins' OR 'hmg-coa statin' OR 'hmg-coa statins' OR 'hmg coa reductase inhibitor' OR 'hmg coa reductase inhibitors' OR 'hmg-coa reductase inhibitors' OR 'hydroxymethylglutaryl coa inhibitor' OR 'hydroxymethylglutaryl coa inhibitors' OR 'hydroxymethylglutaryl-coa inhibitor' OR 'hydroxymethylglutaryl-coa inhibitors' OR 'hydroxymethylglutaryl coenzyme a inhibitor' OR 'hydroxymethylglutaryl coenzyme a inhibitors' OR 'hydroxymethylglutaryl-coenzyme a inhibitor' OR 'hydroxymethylglutaryl-coenzyme a inhibitors' OR 'hydroxymethylglutarylcoenzyme a reductase inhibitors' OR 'hydroxymethylglutaryl coa reductase inhibitor' OR 'hydroxymethylglutaryl coa reductase inhibitors' OR 'hydroxymethylglutaryl-coa reductase inhibitor' OR 'hydroxymethylglutaryl-coa reductase inhibitors' OR 'hmgcr inhibitor' OR 'hmgcr inhibitors' OR 'lipid-lowering drugs' OR 'lipid-lowering agents'
3. #1 AND #2

**Table** **S2. Quality assessment according to Newcastle-Ottawa scale**

|  | **Selection** | | | | **Comparability** | **Outcome (Exposure)** | | |  |
| --- | --- | --- | --- | --- | --- | --- | --- | --- | --- |
| **Author, Year** | Representativeness of the exposed cohort  Is the case definition adequate | Selection of the non exposed cohort  Representativeness of the cases | Ascertainment of exposure  Selection of Controls | Demonstration that outcome of interest was not present at start of study  Definition of Controls | Comparability of cohorts on the basis of the design or analysis  Comparability of cases and controls on the basis of the design or analysis | Assessment of outcome  Ascertainment of exposure | Was follow-up long enough for outcomes to occur Same method of ascertainment for cases and controls | Adequacy of follow up of cohorts  Non-Response rate | **Total score** |
| Alamdari et al. (2020) [1] | * | * | * | * |  | * | * | * | 7 |
| Argenziano et al. (2020) [2] | * | * | * | * |  | * | * | * | 7 |
| Cariou Bertrand et al. (2020) [3] | * | * |  | * |  | * | * | * | 6 |
| Cariou et al. (2020) [4] | * | * | * | * | ** |  | * | * | 8 |
| Daniels et al. (2020) [5] | * | * |  | * | ** | * | * | * | 8 |
| Davoudi-Monfared et al. (2020) [6] | * | * | * | * |  | * | * | * | 7 |
| De Spiegeleer et al. (2020) [7] | * | * | * |  | ** | * | * | * | 8 |
| Dreher et al. (2020) [8] | * | * | * | * |  | * | * | * | 7 |
| Grasselli et al. (2020) [9] | * | * |  | * | ** |  | * | * | 7 |
| Gupta et al. (2021) [10] | * | * | * | * | ** | * | * | * | 9 |
| Higuchi et al. (2021) [11] | * | * | * | * | ** | * | * | * | 9 |
| Israel et al. (2020) [12] | * | * |  | * | ** | * | * | * | 8 |
| Jakob et al. (2021) [13] |  | * | * | * | ** | * | * | * | 8 |
| Mallow et al. (2020) [14] | * | * | * | * | ** | * | * | * | 9 |
| McCarthy et al. (2020) [15] | * | * | * | * |  | * | * | * | 7 |
| Nguyen et al. (2020) [16] | * | * | * | * | ** |  | * | * | 8 |
| Nicholson et al. (2021) [17] | * | * | * | * | ** | * | * | * | 9 |
| Pitscheider et al. (2020) [18] | * | * |  |  | * | * | * | * | 7 |
| Rodriguez-Nava et al. (2020) [19] | * | * |  | * | ** |  | * | * | 7 |
| Rossi et al. (2020) [20] |  | * |  | * | ** |  | * | * | 6 |
| Saeed et al. (2020) [21] | * | * | * | * | ** |  | * | * | 8 |
| Song et al. (2020) [22] | * | * | * | * | ** | * | * | * | 9 |
| Tan et al. (2020) [23] | * | * | * |  | ** |  | * | * | 7 |
| Wang et al. (2020) [24] | * | * | * | * | ** |  | * | * | 8 |
| Yan et al. (2020) [25] | * | * | * | * | ** |  | * | * | 8 |
| Yang et al. (2020) [26] | * | * | * | * |  | * | * | * | 7 |
| Zenga et al. (2020) [27] | * | * | * | * | ** |  | * | * | 8 |
| Zhang et al. (2020) [28] | * | * | * | * | ** |  | * | * | 8 |

**Table S3.** PRISMA checklist

| **Section/topic** | **#** | **Checklist item** | **Reported on page #** |
| --- | --- | --- | --- |
| **TITLE** | | |  |
| Title | 1 | Identify the report as a systematic review, meta-analysis, or both. | 1 |
| **ABSTRACT** | | |  |
| Structured summary | 2 | Provide a structured summary including, as applicable: background; objectives; data sources; study eligibility criteria, participants, and interventions; study appraisal and synthesis methods; results; limitations; conclusions and implications of key findings; systematic review registration number. | 3-4 |
| **INTRODUCTION** | | |  |
| Rationale | 3 | Describe the rationale for the review in the context of what is already known. | 6-7 |
| Objectives | 4 | Provide an explicit statement of questions being addressed with reference to participants, interventions, comparisons, outcomes, and study design (PICOS). | 6-7 |
| **METHODS** | | |  |
| Protocol and registration | 5 | Indicate if a review protocol exists, if and where it can be accessed (e.g., Web address), and, if available, provide registration information including registration number. | no apply |
| Eligibility criteria | 6 | Specify study characteristics (e.g., PICOS, length of follow-up) and report characteristics (e.g., years considered, language, publication status) used as criteria for eligibility, giving rationale. | 7-8 |
| Information sources | 7 | Describe all information sources (e.g., databases with dates of coverage, contact with study authors to identify additional studies) in the search and date last searched. | 7-9 |
| Search | 8 | Present full electronic search strategy for at least one database, including any limits used, such that it could be repeated. | 7-9 |
| Study selection | 9 | State the process for selecting studies (i.e., screening, eligibility, included in systematic review, and, if applicable, included in the meta-analysis). | 8-10 |
| Data collection process | 10 | Describe method of data extraction from reports (e.g., piloted forms, independently, in duplicate) and any processes for obtaining and confirming data from investigators. | 7-10 |
| Data items | 11 | List and define all variables for which data were sought (e.g., PICOS, funding sources) and any assumptions and simplifications made. | 7-10 |
| Risk of bias in individual studies | 12 | Describe methods used for assessing risk of bias of individual studies (including specification of whether this was done at the study or outcome level), and how this information is to be used in any data synthesis. | 7-10 |
| Summary measures | 13 | State the principal summary measures (e.g., risk ratio, difference in means). | 7-10 |
| Synthesis of results | 14 | Describe the methods of handling data and combining results of studies, if done, including measures of consistency (e.g., I^2^) for each meta-analysis. | 7-10 |

| **Section/topic** | **#** | **Checklist item** | **Reported on page #** |
| --- | --- | --- | --- |
| Risk of bias across studies | 15 | Specify any assessment of risk of bias that may affect the cumulative evidence (e.g., publication bias, selective reporting within studies). | 7-9 |
| Additional analyses | 16 | Describe methods of additional analyses (e.g., sensitivity or subgroup analyses, meta-regression), if done, indicating which were pre-specified. | 7-9 |
| **RESULTS** | | |  |
| Study selection | 17 | Give numbers of studies screened, assessed for eligibility, and included in the review, with reasons for exclusions at each stage, ideally with a flow diagram. | 10-13 |
| Study characteristics | 18 | For each study, present characteristics for which data were extracted (e.g., study size, PICOS, follow-up period) and provide the citations. | 10-13 |
| Risk of bias within studies | 19 | Present data on risk of bias of each study and, if available, any outcome level assessment (see item 12). | 10-13 |
| Results of individual studies | 20 | For all outcomes considered (benefits or harms), present, for each study: (a) simple summary data for each intervention group (b) effect estimates and confidence intervals, ideally with a forest plot. | 10-13 |
| Synthesis of results | 21 | Present results of each meta-analysis done, including confidence intervals and measures of consistency. | 10-13 |
| Risk of bias across studies | 22 | Present results of any assessment of risk of bias across studies (see Item 15). | 10-13 |
| Additional analysis | 23 | Give results of additional analyses, if done (e.g., sensitivity or subgroup analyses, meta-regression [see Item 16]). | 12 |
| **DISCUSSION** | | |  |
| Summary of evidence | 24 | Summarize the main findings including the strength of evidence for each main outcome; consider their relevance to key groups (e.g., healthcare providers, users, and policy makers). | 13-17 |
| Limitations | 25 | Discuss limitations at study and outcome level (e.g., risk of bias), and at review-level (e.g., incomplete retrieval of identified research, reporting bias). | 16-17 |
| Conclusions | 26 | Provide a general interpretation of the results in the context of other evidence, and implications for future research. | 17 |
| **FUNDING** | | |  |
| Funding | 27 | Describe sources of funding for the systematic review and other support (e.g., supply of data); role of funders for the systematic review. | In the title page. |

**Reference**

1. Alamdari NM, Afaghi S, Rahimi FS, et al. Mortality risk factors among hospitalized COVID-19 patients in a major referral center in Iran. Tohoku J Exp Me. 2020;252(1):73-84. https://doi.org/10.1620/tjem.252.73.
2. Argenziano MG, Bruce SL, Slater CL, et al. Characterization and clinical course of 1000 patients with coronavirus disease 2019 in New York: retrospective case series. BMJ. 2020;369:m1996. https://doi.org/10.1136/bmj.m1996.
3. Cariou B, Hadjadj S, Wargny M, et al. Phenotypic characteristics and prognosis of inpatients with COVID-19 and diabetes: the CORONADO study. Diabetologia. 2020;63(8):1500-15. https://doi.org/10.1007/s00125-020-05180-x.
4. Cariou B, Goronflot T, Rimbert A, et al. Routine use of statins and increased mortality related to COVID-19 in inpatients with type 2 diabetes: Results from the CORONADO study. Diabetes Metab. 2020;101202. https://doi.org/10.1016/j.diabet.2020.10.001.
5. Daniels LB, Sitapati AM, Zhang J, et al. Relation of Statin Use Prior to Admission to Severity and Recovery Among COVID-19 Inpatients. Am J Cardiol. 2020;136:149-155. https://doi.org/10.1016/j.amjcard.2020.09.012.
6. Davoudi-Monfared E, Rahmani H, Khalili H, et al. A randomized clinical trial of the efficacy and safety of interferon β-1a in treatment of severe COVID-19. Antimicrob Agents Chemother. 2020;64(9):e01061-20. https://doi.org/10.1128/AAC.01061-20.
7. De Spiegeleer A, Bronselaer A, Teo JT, et al. The Effects of ARBs, ACEis, and Statins on Clinical Outcomes of COVID-19 Infection Among Nursing Home Residents. J Am Med Dir Assoc. 2020;21(7):909-14.e2. https://doi.org/10.1016/j.jamda.2020.06.018.
8. Dreher M, Kersten A, Bickenbach J, et al. The Characteristics of 50 Hospitalized COVID-19 Patients With and Without ARDS. Dtsch Arztebl Int. 2020;117(16):271-8. https://doi.org/10.3238/arztebl.2020.0271.
9. Grasselli G, Greco M, Zanella A, et al. Risk Factors Associated with Mortality among Patients with COVID-19 in Intensive Care Units in Lombardy, Italy. JAMA Intern Med. 2020;180(10):1345-55. https://doi.org/10.1001/jamainternmed.2020.3539.
10. Gupta A, Madhavan MV, Poterucha TJ, et al. Association Between Antecedent Statin Use and Decreased Mortality in Hospitalized Patients with COVID-19. Nat Commun. 2021;12(1):1325. https://doi.org/10.1038/s41467-021-21553-1.
11. Higuchi T, Nishida T, Iwahashi H, et al. Early Clinical Factors Predicting the Development of Critical Disease in Japanese Patients with COVID-19: A Single-Center, Retrospective, Observational Study. J Med Virol. 2021;93(4):2141-8. https://doi.org/10.1002/jmv.26599.
12. Israel A, Schaffer A, Cicurel A, et al. Large population study identifies drugs associated with reduced COVID-19 severity. medRxiv. [cited 2021 April 8]. https://doi.org/10.1101/2020.10.13.20211953.
13. Jakob CEM, Borgmann S, Duygu F, et al. First results of the "Lean European Open Survey on SARS-CoV-2-Infected Patients (LEOSS)". Infection. 2021;49(1):63-73. https://doi.org/10.1007/s15010-020-01499-0.
14. Mallow PJ, Belk KW, Topmiller M, Hooker EA. Outcomes of Hospitalized COVID-19 Patients by Risk Factors: Results from a United States Hospital Claims Database. J Health Econ Outcomes Res. 2020;7(2):165-74. https://doi.org/10.36469/jheor.2020.17331.
15. McCarthy CP, Murphy S, Jones-O'Connor M, et al. Early clinical and sociodemographic experience with patients hospitalized with COVID-19 at a large American healthcare system. EClinicalMedicine. 2020;26:100504. https://doi.org/ 10.1016/j.eclinm.2020.100504.
16. Nguyen AB, Upadhyay GA, Chung B, et al. Outcomes and cardiovascular comorbidities in a predominantly african-american population with COVID-19. medRxiv. [cited 2021 April 8]. https://doi.org/10.1101/2020.06.28.20141929
17. Nicholson CJ, Wooster L, Sigurslid HH, et al. Estimating Risk of Mechanical Ventilation and Mortality Among Adult COVID-19 patients Admitted to Mass General Brigham: The VICE and DICE Scores. EClinicalMedicine. 2021;33:100765. https://doi.org/10.1016/j.eclinm.2021.100765.
18. Pitscheider L, Karolyi M, Burkert FR, et al. (2020) Muscle involvement in SARS-CoV-2 infection. Eur J Neurol. [cited 2021 April 8]. https://doi.org/10.1111/ene.14564.
19. Rodriguez-Nava G, Trelles-Garcia DP, Yanez-Bello MA, Chung CW, Trelles-Garcia VP, Friedman HJ. Atorvastatin associated with decreased hazard for death in COVID-19 patients admitted to an ICU: A retrospective cohort study. Crit Care. 2020;24(1):429. https://doi.org/10.1186/s13054-020-03154-4.
20. Rossi R, Talarico M, Coppi F, Boriani G. Protective role of statins in COVID 19 patients: importance of pharmacokinetic characteristics rather than intensity of action. Intern Emerg Med. 2020;15(8):1573-6. https://doi.org/10.1007/s11739-020-02504-y.
21. Saeed O, Castagna F, Agalliu I, et al. Statin Use and In-Hospital Mortality in Diabetics with COVID-19. J Am Heart Assoc. 2020;9(24):e018475. https://doi.org/10.1161/JAHA.120.018475.
22. Song SL, Hays SB, Panton CE, et al. Statin Use Is Associated with Decreased Risk of Invasive Mechanical Ventilation in COVID-19 Patients: A Preliminary Study. Pathogens. 2020;9(9):759. https://doi.org/10.3390/pathogens9090759.
23. Tan WYT, Young BE, Lye DC, Chew DEK, Dalan R. Statin use is associated with lower disease severity in COVID-19 infection. Sci Rep. 2020;10(1):17458. https://doi.org/10.1038/s41598-020-74492-0.
24. Wang B, Van Oekelen O, Mouhieddine TH, et al. A tertiary center experience of multiple myeloma patients with COVID-19: Lessons learned and the path forward. J Hematol Oncol. 2020;13(1):94. https://doi.org/10.1186/s13045-020-00934-x.
25. Yan H, Valdes AM, Vijay A, et al. Role of Drugs Used for Chronic Disease Management on Susceptibility and Severity of COVID-19: A Large Case-Control Study. Clin Pharmacol Ther. 2020;108(6):1185-94. https://doi.org/10.1002/cpt.2047.
26. Yang D, Xiao Y, Chen J, et al. COVID-19 & Chronic Renal Disease: Clinical characteristics & prognosis. QJM. 2020;113(11):799-805. https://doi.org/10.1093/qjmed/hcaa258.
27. Zenga H, Zhang T, He X, et al. Impact of Hypertension on Progression and Prognosis in Patients with COVID-19 A Retrospective Cohort Study in 1031 Hospitalized Cases in Wuhan, China. medRxiv. [cited 2021 April 8]. https://doi.org/10.1101/2020.06.14.20125997
28. Zhang XJ, Qin JJ, Cheng X, et al. In-Hospital Use of Statins Is Associated with a Reduced Risk of Mortality among Individuals with COVID-19. Cell Metab. 2020;32(2):176-87.e174. https://doi.org/10.1016/j.cmet.2020.06.015.
